# Protein Kinase C**α** Modulates Estrogen-Receptor-Dependent Transcription and Proliferation in Endometrial Cancer Cells

**DOI:** 10.1155/2013/537479

**Published:** 2013-06-17

**Authors:** Alicia M. Thorne, Twila A. Jackson, Van C. Willis, Andrew P. Bradford

**Affiliations:** ^1^Department of Obstetrics and Gynecology, School of Medicine, University of Colorado Anschutz Medical Campus, Aurora, CO 80045, USA; ^2^Division of Rheumatology, Department of Medicine, School of Medicine, University of Colorado Anschutz Medical Campus, Aurora, CO 80045, USA

## Abstract

Endometrial cancer is the most common invasive gynecologic malignancy in developed countries. The most prevalent endometrioid tumors are linked to excessive estrogen exposure and hyperplasia. However, molecular mechanisms and signaling pathways underlying their etiology and pathophysiology remain poorly understood. We have shown that protein kinase C**α** (PKC**α**) is aberrantly expressed in endometrioid tumors and is an important mediator of endometrial cancer cell survival, proliferation, and invasion. In this study, we demonstrate that expression of active, myristoylated PKC**α** conferred ligand-independent activation of estrogen-receptor- (ER-) dependent promoters and enhanced responses to estrogen. Conversely, knockdown of PKC**α** reduced ER-dependent gene expression and inhibited estrogen-induced proliferation of endometrial cancer cells. The ability of PKC**α** to potentiate estrogen activation of ER-dependent transcription was attenuated by inhibitors of phosphoinositide 3-kinase (PI3K) and Akt. Evidence suggests that PKC**α** and estrogen signal transduction pathways functionally interact, to modulate ER-dependent growth and transcription. Thus, PKC**α** signaling, via PI3K/Akt, may be a critical element of the hyperestrogenic environment and activation of ER that is thought to underlie the development of estrogen-dependent endometrial hyperplasia and malignancy. PKC**α**-dependent pathways may provide much needed prognostic markers of aggressive disease and novel therapeutic targets in ER positive tumors.

## 1. Introduction

Endometrial cancer is the most common invasive gynecological malignancy in the United States, accounting for 45,000 new cancer cases and over 7,500 deaths annually [1]. However, molecular mechanisms underlying its etiology and pathophysiology are poorly understood. Endometrial carcinomas are derived from glandular epithelium and typically divided into two subtypes based on clinical, histological, and molecular characteristics [2, 3]. Type I tumors, comprising 80% of cases, are generally well or moderately differentiated with endometrioid morphology and are associated with chronic unopposed estrogen exposure and hyperplasia. By contrast, type II tumors are more heterogeneous, poorly differentiated and may be estrogen independent, arising in a background of atrophic endometrium [2, 4]. The prevalence of advanced stage, high-grade tumors, of both types, with recurrent metastatic disease is increasing [5, 6]. Such cancers typically have a poorer prognosis and are refractory to current therapeutic regimens [7].

 Endometrioid tumors retain expression of estrogen (ER) and progesterone (PR) receptors [8], and estrogen is a critical regulator of endometrial proliferation [9, 10]. Indeed, the majority of endometrial cancers are thought to arise due to unopposed estrogen action leading to hyperplasia and malignant transformation [2, 11]. However, our understanding of the molecular mechanisms underlying the pathophysiology of endometrial cancer lags far behind that of other hormone-dependent malignancies such as breast, prostate and ovarian cancer [2, 8, 12, 13].

The protein kinase C (PKC) family has been implicated in the regulation of numerous signal transduction pathways, modulating cell growth, differentiation, and survival [14–16]. In endometrial cancer cells and primary endometrial epithelium, expression of PKC*α* is increased in response to treatment with estrogen and tamoxifen and may underlie the proliferative actions of these agents in the endometrium [17, 18]. We have previously shown that PKC*α* is aberrantly expressed in human endometrial tumors [19, 20] and is a critical regulator of endometrial cancer cell survival, proliferation, transformation, invasion, and response to chemotherapy [21, 22]. In addition, we demonstrated that knockdown of PKC*α* inhibits growth of estrogen-dependent endometrial cancers in an *in vivo* model [20].

 In this study, we present evidence that, in type I endometrial cancer cells, PKC*α* induces hormone-independent activation of ER, potentiates estrogen transcriptional responses, and regulates estrogen-dependent proliferation and gene expression. Thus, PKC*α* signaling may be a critical element of the supraphysiologic activation of ER thought to underlie the development of endometrial hyperplasia and malignancy.

## 2. Materials and Methods

### 2.1. Cell Lines

Ishikawa and HEC-50 endometrial carcinoma cells were a generous gift from Dr. Leslie (University of Iowa). Ishikawa cells expressing luciferase (luc) or PKC*α* shRNAs have been described [21]. Unless stated otherwise, all cell lines were maintained in 5% CO_2_, phenol red free DMEM, supplemented with charcoal stripped 10% fetal bovine serum, 10 units/mL penicillin, 10 *μ*g/mL streptomycin, and 200 *μ*M L-glutamine. Prior to estrogen treatment (100 nM Estradiol, Sigma Aldrich, St. Louis, MO, USA), cells were transferred to phenol red free DMEM containing 1x SR-1 serum replacement (Sigma Aldrich, St. Louis, MO, USA). Cell lines used were authenticated by analysis of DNA microsatellite short tandem repeats (STRS), as described previously [23].

### 2.2. Cell Proliferation

Cell number and viability were determined from subconfluent cultures using a Vi-Cell Coulter Counter (Beckman-Coulter, Inc., Fullerton, CA, USA) as described in [20].

### 2.3. Luciferase Reporter Assays

The ERE-luc and pS2-luc promoter reporter constructs have been described in [24–26]. Myristoylated PKC*α* vector [27] was obtained from Addgene (Cambridge, MA). Cells (2.0 × 10^5^) were transiently transfected with 0.5 *μ*g ERE-Luc or pS2-luc reporter plasmids using Lipofectamine 2000 (Invitrogen, Carlsbad, CA, USA) as per the manufacturers protocol. 0.5 *μ*g pCMV*β*, encoding *β*-galactosidase under control of the CMV constitutive promoter, was included as a control for transfection efficiency and cell number. Total DNA was kept constant by addition of empty vectors. Promoter activity was determined by Luciferase and *β*-galactosidase assays, as described in [28].

### 2.4. RNA Isolation and Quantitative RTPCR

RNA was isolated from 10^6^ cells using a Qiagen RNeasy kit (Qiagen, Germantown, MD, USA) according to the manufacturer's directions and quantitated using a NanoDrop ND1000 spectrophotometer. Aliquots were evaluated by chromatography using an Agilent RNA 6000 Nano LabChip kit (Agilent Technologies, Santa Clara, CA, USA) on an Agilent Bioanalyzer 2100 system. cDNAs were prepared using iScript cDNA synthesis kit (Bio-Rad, Hercules, CA, USA) as per the manufacturers instructions. The samples were amplified by real-time PCR using iQ SYBR green supermix (Bio-Rad, Hercules, CA, USA) on a Bio-Rad CFX96 C1000 Thermal Cycler using the following conditions: 10 minutes at 95°C and 40 cycles of 15 seconds at 95°C and 1 minute at 60°C. Negative control RNA samples were not reverse transcribed or did not lack PCR template. Results were analyzed with qbase^PLUS^ software (Bio-Rad Hercules, CA, USA), and changes in expression, relative to *β*-actin and rpl13a controls, were estimated using the ΔCT method [29]. Primer pair sequences (forward and reverse, 5′ to 3′) were as follows: *β*-Actin: AGCCTCGCCTTTGCCGA and GCGCGGCGATATCATCATC; RPL13A: TACCAGAAAGTTTGCTTACGTGGG and TGCCTGTTTCCGTAACCTCAAG; PRKCA: GCTTCCAGTGCCAAGTTTGC and GCACCCGGACAAGAAAAAGTAA; LTF: ATGGTGGTTTCATATACGAGGCA and GCCACGGCATAATAGTGAGTT; c-FOS: AAAAGGAGAATCCGAAGGGAAA and GTCTGTCTCCGCTTGGAGTGTAT; pS2 (TFF1): AGGCCCAGACAGAGACGTGTAC and CGTCGAAACAGCAGCCCTTA. Primers were designed using Primer3 software (http://primer3.wi.mit.edu) and obtained from Eurofins MWG Operon (Huntsville, AL, USA) or Integrated DNA Technologies (Coralville, IA, USA).

### 2.5. Statistical Analysis

Data were expressed as mean ± standard deviation or standard error of the mean and analyzed using Student's *t*-test. *P* values <0.05 were considered significantly different.

## 3. Results

**Figure 1:**
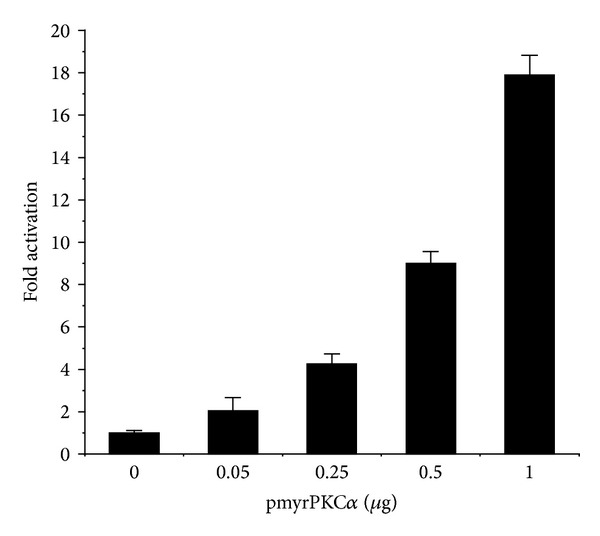
PKC*α* activates an estrogen responsive promoter. Ishikawa cells were transiently transfected with 0.5 *μ*g pEREluc, 0.3 *μ*g pCMV*β*, and the indicated amounts of pmyrPKC*α* or vector control (pCDNA3). Luciferase activity was normalized to *β*-galactosidase and promoter activity expressed as fold increase over control. Data are mean ± s.d. (*n* = 3).

**Figure 2:**
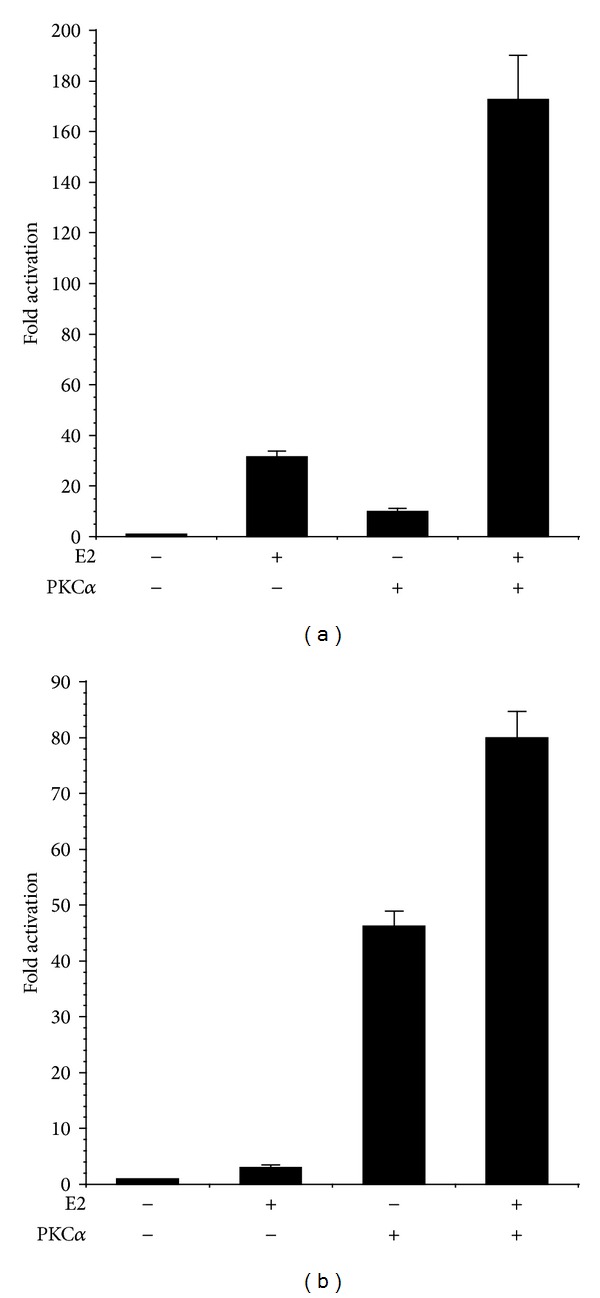
PKC*α* enhances ER-dependent promoter activity. Ishikawa cells were transiently transfected with (a) 0.5 *μ*g pEREluc or (b) 0.5 *μ*g pPS2luc and 0.3 *μ*g pCMV*β* in the presence or absence of 0.5 *μ*g pmyrPKC*α* or vector control (pCDNA3). Cells were treated with ±100 nM estradiol (E2), as indicated. Luciferase activity was normalized to *β*-galactosidase and promoter activity expressed as fold increase over control. Data are mean ± s.e.m of 6 experiments conducted in triplicate.

 To investigate the functional role of PKC*α* signal transduction in the regulation of ER-dependent transcription, Ishikawa endometrial cancer cells were transiently transfected with a myristoylated PKC*α* construct (myrPKC*α*) that is targeted to membranes and thereby rendered constitutively active [21, 27]. As shown in Figure 1, expression of myrPKC*α*, in the absence of estrogen, resulted in a dose-dependent activation of transcription from a promoter containing 3 copies of a canonical estrogen response element (ERE) fused to luciferase [30]. Treatment of Ishikawa cells with estradiol (E2) increased the activity of the ERE promoter approximately 30-fold (Figure 2(a)). In the presence of activated myrPKC*α*, E2-stimulated ERE promoter activity was further increased over 170-fold. Thus, PKC*α* induced hormone-independent activity of an ERE and potentiated the effect of estrogen. Similar results were obtained using the pS2 (TFF1) promoter, an endogenous E2 regulated gene [31] (Figure 2(b)). myrPKC*α* expression induced a marked increase in basal pS2 promoter activity and enhanced the stimulatory effect of E2. Treatment with E2 had no effect on the level of myrPKC*α* expression in Ishikawa cells (not shown).

**Figure 3:**
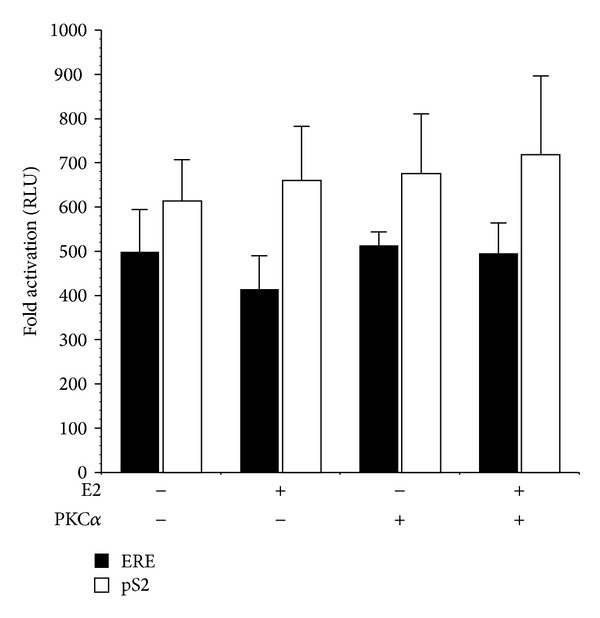
Estrogen and PKC*α* responses are ER dependent. HEC-50 cells, lacking ER, were transiently transfected with 0.5 *μ*g pEREluc or 0.5 *μ*g pPS2luc and 0.3 *μ*g pCMV*β* in the presence or absence of 0.5 *μ*g pmyrPKC*α* or vector control (pCDNA3). Cells were treated with ±100 nM estradiol (E2), as indicated. Luciferase activity was normalized to *β*-galactosidase and promoter activity expressed as Relative Light Units (RLU). Data are mean ± s.e.m of 4 experiments conducted in triplicate.

**Figure 4:**
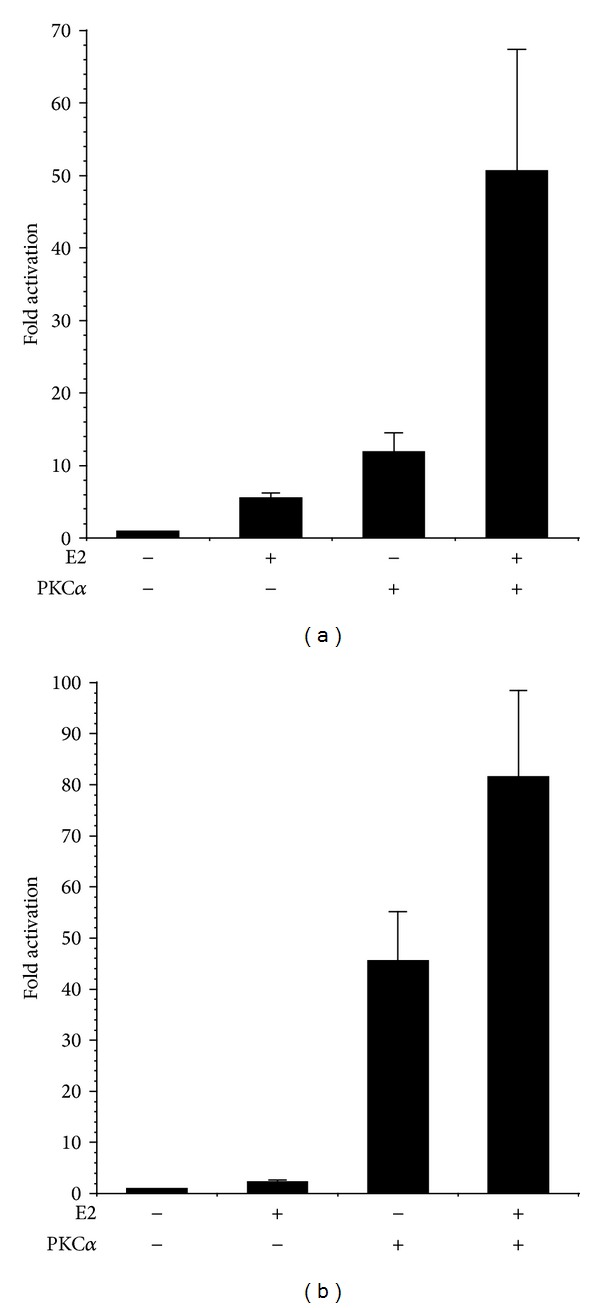
Reconstitution of PKC*α* regulated, ER-dependent transcription in HEC-50 cells. Cells were transiently transfected with 0.5 *μ*g pHEGO (ER*α*) and (a) 0.5 *μ*g pEREluc or (b) 0.5 *μ*g pPS2luc and 0.3 *μ*g pCMV*β* in the presence or absence of 0.5 *μ*g pmyrPKC*α* or vector control (pCDNA3). Cells were treated with ±100 nM estradiol (E2), as indicated. Promoter activity was determined as in Figure 2. Data are mean ± s.e.m of 6 experiments conducted in triplicate.

 In HEC-50 endometrial cancer cells, which lack estrogen receptor (ER) [32], activity of the ERE and pS2 promoters was minimal (Figure 3). Expression of active PKC*α* or treatment with E2 (in the presence or absence of myrPKC*α*) had no effect on pS2 or ERE promoter activity, indicating that the effects of PKC*α* and E2 are dependent on ER expression (Figure 3). Accordingly, transfection of HEC-50 cells with pHEGO encoding ER*α* reconstituted ERE and pS2 transcriptional responses to both E2 and myrPKC*α* (Figure 4). Expression of ER*α* in HEC-50 cells also restored the enhancement of E2-stimulated promoter activity by PKC*α*. (Figure 4). Together, these results (Figures 1–4) indicate that PKC*α* signaling induces ligand-independent activation of ER-dependent transcription and thereby potentiates responses to E2.

**Figure 5:**
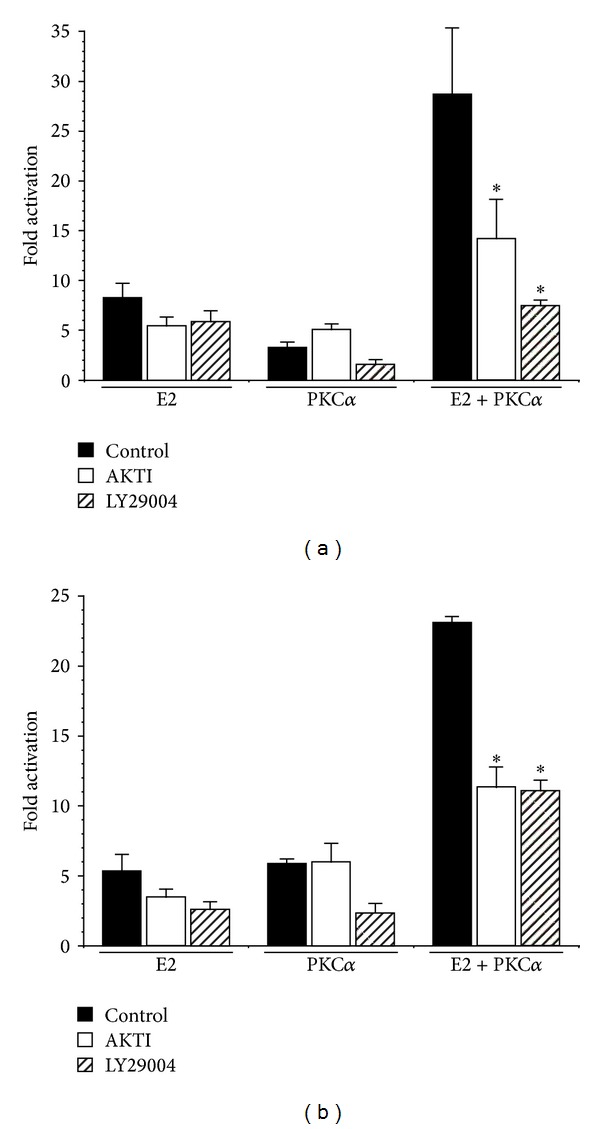
PKC*α* effects on ER-dependent transcription are mediated by the PI3-kinase/Akt pathway. (a) Ishikawa cells were transiently transfected with 0.5 *μ*g pEREluc and 0.3 *μ*g pCMV*β*, in the presence or absence of 0.5 *μ*g pmyrPKC*α*. (b) HEC-50 cells were transiently transfected with ER*α* (0.5 *μ*g pHEGO), 0.5 *μ*g pEREluc, and 0.3 *μ*g pCMV*β* ± 0.5 *μ*g pmyrPKC*α* or pCDNA3. Cells were treated with ±100 nM estradiol (E2) in the presence or absence of the Akt and PI3K inhibitors, Akt-I-1/2 (1 *μ*M) and LY29004 (10 *μ*M), respectively. Promoter activity was determined as in Figure 2 and expressed as fold increase over the appropriate inhibitor or diluent control. Results are mean ± s.d. (*n* = 6). *P < 0.05.

 Activation of the phosphoinositide 3-kinase (PI3K)/Akt pathway is one of the most critical steps in endometrial carcinogenesis [11] and has been shown to mediate ligand-independent activation of ER [33, 34]. Moreover, we have previously implicated PKC*α* in the regulation of Akt in endometrial cancer cells [22]. To investigate the role of PI3K/Akt signaling in PKC*α* regulation of transcription, we treated Ishikawa cells with pharmacological inhibitors of PI3K (LY 29004) or Akt (Akt-I-1/2) [35, 36] and examined their effects on the ERE promoter (Figure 5). Treatment of Ishikawa cells with LY29004 or Akt-I-1/2 significantly inhibited the ability of myrPKC*α* to enhance E2 activation of the ERE promoter (Figure 5(a)). Similar results were obtained in HEC-50 cells transfected with ER*α* (Figure 5(b)). LY29004 and Akt-I-1/2 treatment resulted in the expected decrease in phosphorylation of Akt and GSK3, respectively, and did not impact expression of myrPKC*α* (not shown). Thus, the effects of PKC*α* on E2- and ER-dependent transcription are mediated, in part, by the PI3K/Akt pathway.

**Figure 6:**
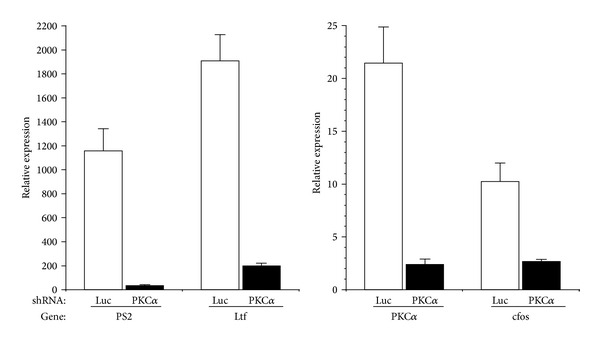
Knockdown of PKC*α* reduces ER-dependent gene expression. Ishikawa cells were stably transduced with shRNAs targeting PKC*α* or luciferase (luc). RNA was isolated and analyzed by real time reverse transcription PCR, using primers specific for the indicated gene, as described in Section 2. ΔCt values were calculated relative to a control gene (rp13) and relative levels expressed as 2^Δct^. Data are mean ± s.e.m (*n* = 6).

 To confirm the results, using the ERE and pS2 promoter constructs, we examined expression of a panel of estrogen-dependent genes implicated in endometrial neoplastic transformation [33, 34]. Levels of pS2 (TFF1), lactotransferrin (Ltf), and c-fos mRNA were determined by real-time reverse transcription PCR, in Ishikawa cells stably expressing shRNA to knockdown PKC*α*. Control cells were transduced with shRNA targeting luciferase [20]. As shown in Figure 6, knockdown of PKC*α* in Ishikawa cells significantly reduced expression of the estrogen-dependent genes pS2, ltf, and c-fos. PKC*α* shRNA expressing cells also exhibited the expected decrease in PKC*α* mRNA levels (Figure 6). 

**Figure 7:**
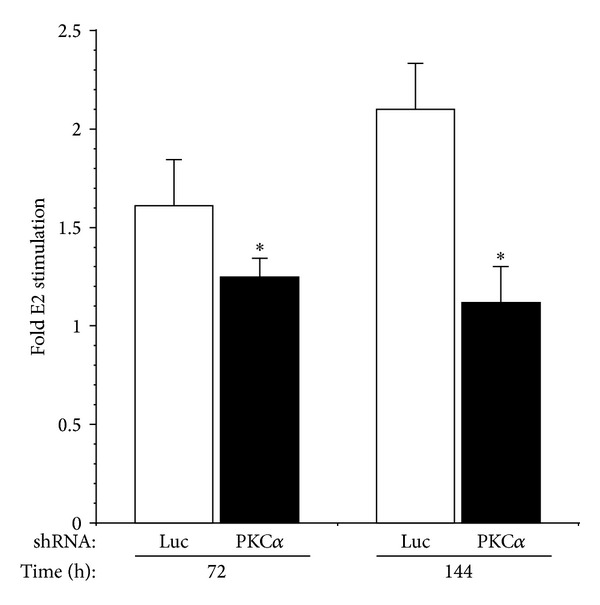
Knockdown of PKC*α* inhibits estrogen-stimulated growth of Ishikawa cells. Cells were stably transfected with shRNAs targeting PKC*α* or luciferase. Control (luc) or PKC*α* knockdown cell lines were treated with ±100 nM estrogen (E2) and harvested at the indicated time points. Cell number and viability were determined using a Beckmann Coulter Vi-CELL analyzer. Results are expressed as the fold increase in cell number induced by estrogen treatment. Data are mean ± s.e.m (*n* = 6). *P ≤ 0.05.

Estrogen is a critical regulator of type I endometrial cancer growth and stimulates proliferation of Ishikawa cells [9, 37–39]. We therefore determined the effect of PKC*α* knockdown on estrogen-dependent proliferation. E2 treatment stimulated proliferation of Ishikawa cells expressing a control shRNA targeting luciferase, reflected by an increase in the number of viable cells (Figure 7). Knockdown of PKC*α* significantly reduced the E2-dependent increase in cell number at 72 h and essentially abrogated the E2 proliferative response at 144 h. Cell viability (89%–96%) was not significantly different between cell lines and was not affected by E2 treatment. 

Together, these results indicate that PKC*α* is a critical regulator of ER-dependent gene expression and modulates both E2-stimulated transcription and cell proliferation in ER positive endometrial cancer cells.

## 4. Discussion

Estrogen, acting through ER, is a major contributor to endometrial proliferation. Indeed, hormone-dependent, type I endometrial cancers are thought to arise due to excess estrogen stimulation, unopposed by progesterone, promoting mitogenesis, atypical hyperplasia, and the transition to malignant adenocarcinoma [4, 8, 11]. In this study, we have shown that activation of PKC*α* is a critical element of such an estrogenic environment, resulting in estrogen-independent activation of ER-dependent transcription and potentiating the effects of estrogen on both gene expression and endometrial cancer cell proliferation. The primary effect of PKC*α* is to stimulate basal, unliganded ER transactivation, thereby amplifying estrogen-stimulated promoter activity and enhancing levels of genes linked to endometrial hyperplasia and malignancy. 

 To confirm the observed interaction of PKC*α* and ER signaling on estrogen responsive promoters, we examined levels of a subset of estrogen responsive genes (lactotransferrin, pS2/TFF1, and c-fos) implicated in proliferation of normal and transformed endometrial cells and linked to the development of endometrial carcinoma [11, 33, 34, 40, 41]. Knockdown of PKC*α* in endometrial cancer cells reduced expression of these genes (Figure 6) consistent with their regulation by both ER and PKC*α*. Accordingly, treatment of breast and endometrial cancer cells with phorbol esters, to activate PKC, has been shown to induce expression of pS2 and c-fos and augment their increased levels observed in response to estrogen treatment [41–43]. 

 Cyclin D1 is also an important mediator of estrogen-dependent endometrial cell proliferation and is over expressed in endometrioid tumors [9, 37]. Consistent with interaction of E2 and PKC*α* mitogenic signaling pathways, we previously demonstrated that PKC*α* activates the cyclin D1 promoter in endometrial cancer cells [20]. In addition, expression of the cyclin-dependent kinase (CDK) inhibitor p21 is decreased in endometrial cancers, correlating with poorer prognosis [44, 45]. Estrogen-induced Ishikawa cell proliferation paralleled a decline in p21 protein expression [9], whilst progesterone mediated growth inhibition was linked to elevated p21 levels [46]. Expression of p21 was also upregulated in response to knockdown of PKC*α* [20], suggesting that the CDK inhibitor is a target of both PKC*α* and estrogen signaling pathways, regulating endometrial cancer cell proliferation.

 The PI3K/Akt pathway is commonly dysregulated in type I endometrial cancers. More than 80% of endometrioid carcinomas exhibit loss of the tumor suppressor PTEN and/or activating mutations in PI3K [47–49]. PTEN heterozygous mice develop endometrial hyperplasia and adenocarcinoma, characteristic of human endometrioid tumors [11, 33, 34]. Endometrial tumorigenesis in this model is associated with upregulation of estrogen-stimulated gene expression and ligand-independent activation of ER [34], mediated by Akt [33]. Consistent with these results, we have shown that PKC*α* is required to maintain Akt activity in endometrial cancer cells [20] and that amplification of estrogen/ER mediated transcription by PKC*α* is dependent upon the PI3K/Akt pathway (Figure 5). 

Phosphorylation of ER has been implicated in regulation of its transcriptional activity and DNA binding [50, 51]. Phosphorylation of serine 167, by Akt, induces activation of ER [33], and phosphorylation of serines 104, 106, and 118 modulates ER interaction with co activators [52]. PKC*α*-dependent ER phosphorylation and its functional role in endometrial cancer cells remain to be established; however, these latter sites match the consensus substrate sequence for PKC and, since PKC*α* regulates Akt activity [20], suggest that the effects of PKC*α* may be mediated by direct or indirect phosphorylation of ER.

## 5. Conclusions

In summary, we have shown that activation of PKC*α* induces estrogen-independent activation of ER-dependent gene expression and potentiates the effects of estrogen on transcription. Evidence also implicates PKC*α* in the regulation of estrogen-dependent endometrial cancer cell proliferation. Thus, PKC*α*-dependent signal transduction is a critical component of the environment of excessive estrogen and supraphysiologic activation of ER, which is thought to underlie the development of endometrial hyperplasia and endometrioid adenocarcinoma. Furthermore, estrogen exposure may increase PKC*α* expression and/or activity in endometrial cancer cells [17, 18, 53], providing a potential positive feedback loop to amplify estrogen and ER-dependent responses.

 The incidence of endometrial cancer continues to rise, and, despite advances in hormonal and chemotherapy, overall survival has not significantly improved [54–56]. Thus, there is an evident need to develop novel, molecular targeted therapies. PKC*α* is a critical element in the estrogen, PI3K/Akt, and growth factor/ERK-dependent signal transduction pathways regulating the growth of type I tumors [20–22]. Hence, inhibition of PKC*α*-dependent signaling would enable the simultaneous targeting of multiple estrogen-dependent and -independent pathways implicated in the development and progression of endometrial carcinogenesis. PKC*α* specific inhibitors [57–59] may provide novel avenues, for primary or adjunct therapeutic intervention, to target tumors resistant to current regimens.
